# Supplementary material for: White LED Lighting Increases the Root Productivity of Panax ginseng C. A. Meyer in a Hydroponic Cultivation System of a Plant Factory
Source: Biology (Basel). 2023 Jul 26;12(8):1052. doi: 10.3390/biology12081052 (PMC10452227; doi:10.3390/biology12081052)
Supplement: Supplementary file 1 [file biology-12-01052-s001.zip › biology-2494864-supplementary.pdf]

Supplementary Materials

Table S1. Model fit of physiological and growth models of *Panax ginseng* according to light environment.

	AIC	BIC	n	Baseline test		
				χ^2	df	p
Model	447.278	523.759	50	23.686	11	0.014

Table S2. Additional fit measure results.

**Index**	**Value**
Comparative Fit Index (CFI)	0.973
Normed fit index(NFI)	0.953
Bollen's Incremental Fit Index (IFI)	0.974
Relative Noncentrality Index (RNI)	0.973
Goodness of fit index (GFI)	**0.993**

Table S3. Regression coefficients and p-values of model.

Predictor		Outcome	Estimate	Std. Error	z-value	p	**Standardized**		
							All	LV	Endo
E	~	ADW	0.060	0.154	0.392	0.695	-0.241	0.362	0.061
R	~	ADW	0.900	0.289	3.113	0.002	0.333	1.467	0.448
Y	~	ADW	1.738	0.445	3.902	< .001	0.865	2.611	0.706
Ci	~	RDW	0.467	0.128	3.643	< .001	0.216	0.718	0.465
B	~	RDW	-0.942	0.280	-3.369	< .001	-1.490	-0.394	-0.465
W	~	RDW	1.167	0.306	3.813	< .001	0.567	1.767	0.470
R	~	Pn	-0.505	0.292	-1.728	0.084	-1.078	0.068	-0.250
B	~	Pn	0.065	0.292	0.222	0.824	-0.508	0.638	0.032
W	~	Pn	1.925	0.293	6.565	< .001	1.351	2.500	0.778
Y	~	Pn	-0.360	0.459	-0.785	0.433	-1.259	0.539	-0.145
gs	~	Pn	0.017	0.122	0.141	0.888	-0.221	0.255	0.017
R	~	E	-0.134	0.037	-3.587	< .001	-0.207	-0.061	-0.066
B	~	E	-0.072	0.036	-1.994	0.046	-0.144	-0.001	-0.036
W	~	E	0.281	0.050	5.646	< .001	0.183	0.379	0.114
Y	~	E	0.145	0.057	2.526	0.012	0.032	0.257	0.059
gs	~	E	0.923	0.015	61.102	< .001	0.894	0.953	0.923

Pn	~	E	-0.041	0.018	-2.342	0.019	-0.076	-0.007	-0.041
R	~	Ci	2.486	0.144	17.208	< .001	2.203	2.769	1.230
B	~	Ci	2.233	0.140	15.897	< .001	1.958	2.508	1.105
W	~	Ci	-1.028	0.194	-5.306	< .001	-1.408	-0.648	-0.416
Y	~	Ci	2.360	0.225	10.473	< .001	1.918	2.802	0.954
Pn	~	Ci	-0.159	0.068	-2.346	0.019	-0.292	-0.026	-0.159
E	~	Ci	0.073	0.063	1.160	0.246	-0.050	0.196	0.073
R	~	gs	0.042	0.340	0.124	0.902	-0.624	0.708	0.021
B	~	gs	-0.021	0.340	-0.062	0.951	-0.687	0.645	-0.010
W	~	gs	0.210	0.340	0.618	0.537	-0.456	0.876	0.085
**Y**	~	**gs**	1.638	0.481	3.407	< .001	0.696	2.581	0.662
**RDW**	**~~**	**ADW**	-0.104	0.091	-1.144	0.252	-0.164	-0.104	-0.164

~: Regression analysis, ~~:Correlation analysis.
